# A novel approach for APT attack detection based on an advanced computing

**DOI:** 10.1038/s41598-024-72957-0

**Published:** 2024-09-27

**Authors:** Cho Do Xuan, Tung Thanh Nguyen

**Affiliations:** 1 Faculty of Information Security, Posts and Telecommunications Institute of Technology, Hanoi, Vietnam; 2 National Institute of Digital Technology and Digital Transformation, Ministry of Information and Communications, Hanoi, Vietnam

**Keywords:** BiLSTM, Attention, Dynamic graph convolutional neural network, APT attack detection, Applied mathematics, Computational science, Computer science

## Abstract

To enhance the effectiveness of the Advanced Persistent Threat (APT) detection process, this research proposes a new approach to build and analyze the behavior profiles of APT attacks in network traffic. To achieve this goal, this study carries out two main objectives, including (i) building the behavior profile of APT IP in network traffic using a new intelligent computation method; (ii) analyzing and evaluating the behavior profile of APT IP based on a deep graph network. Specifically, to build the behavior profile of APT IP, this article describes using a combination of two different data mining methods: Bidirectional Long Short-Term Memory (Bi) and Attention (A). Based on the obtained behavior profile, the Dynamic Graph Convolutional Neural Network (DGCNN) is proposed to extract the characteristics of APT IP and classify them. With the flexible combination of different components in the model, the important information and behavior of APT attacks are demonstrated, not only enhancing the accuracy of detecting attack campaigns but also reducing false predictions. The experimental results in the paper show that the method proposed in this study has brought better results than other approaches on all measurements. In particular, the accuracy of APT attack prediction results (Precision) reached from 84 to 91%, higher than other studies of over 7%. These experimental results have proven that the proposed BiADG model for detecting APT attacks in this study is proper and reasonable. In addition, those experimental results have not only proven the effectiveness and superiority of the proposed method in detecting APT attacks but have also opened up a new approach for other cyber-attack detections such as distributed denial of service, botnets, malware, phishing, etc.

## Introduction

### Problems statement

APT attack is one of the most dangerous network attack techniques nowadays^1,2^. According to the statistics presented in^3,4^, all organizations from any country can become victims of this type of attack. Therefore, detecting and early warning APT attack campaigns are very necessary. To detect APT attacks, current network attack monitoring and detecting systems often rely on analyzing and evaluating the data from various sources such as DNS logs, Network Traffic or weblogs^1^. Network traffic data are the most useful source of information since they contain a lot of important network behaviors^1,2^. The two main APT attack detection approaches based on network traffic^2–5^ include:

- Analyzing network traffic into different components, such as domains, IPs, protocols, etc. Then, APT abnormal behaviors are defined and extracted^6–13^. The advantage of this approach is that APT attack behaviors in the system can be detected fast and accurately. However, researchers in^2^ and ^11^ pointed out some disadvantages of this method, including late detection time, difficulty deploying in real systems, difficulty to define and extract attack behaviors, and lack of experimental data.
- Using analytic tools to extract statistical features of network traffic data. In this approach, APT attacks are detected based on data-sets with available features from network traffic data such as^1,2,14–16,52^: DARPA/KDD Cup99, CAIDA, NSL-KDD, ISCX 2012, UNSW-NB15, etc. Thus, it can be seen that APT attack detection methods in this direction do not directly extract the anomalous behavior but rely on the available features of the data sets. The advantage of this approach is the less computation cost thanks to the use of available features from the data-sets. Nevertheless, this approach has a drawback: The above data-sets used are taken from the laboratory, and do not contain any data about real-life APT attacks, so its available features may not be suitable for detecting APT attacks and cannot detect APT attacks in practice. In addition, redundant features may downgrade the attack detection performance of the system.

In order to overcome the disadvantages of the two approaches above, studies in^11,16–19^ have proposed some APT detection methods based on building behavioral profiles. In these approaches, APT-specific behaviors are not used. Instead, some special attributes of the data are extracted, based on which the behavioral profiles of the attack campaign are constructed. After constructing the attack behavioral profiles, recent studies usually focus on extracting some features using machine learning or deep learning algorithms. Researchers in^1,16,17,19,20^ use graph-based behavioral profiles to detect APT attacks. Such approaches have been shown to be effective due to their ability to synthesize a number of associations related to the behaviors of APT attacks. However, there are two main issues that need to be improved as follows.

- The use of machine learning or deep learning algorithms to synthesize and extract the anomalous behaviors of APT has brought good results, because these models are able to learn and evaluate the attributes. However, the disadvantage of these methods is that they cannot aggregate and highlight the most important properties and behaviors of the attack data. This results in the effectiveness of APT detection systems often being limited.
- Graph-based behavioral profiles are shown to work well for APT attack detection. However, the main disadvantages of this approach are the high computation cost and the difficulty in extracting the relationship between edges in the graph^17,19,20^. In many real APT attack campaigns, attackers often try to use multiple IPs from command and control servers to connect with malware. Therefore, finding out the relationship between IPs helps detail the unusual behavior of IPs.

### Problem solving

- Operating principles of the proposed model.

To address the enumerated issues in section “Problems statement”, we propose a completely new approach for the task of detecting APT attacks based on the technique of building, extracting, and classifying behavioral profiles of each IP in network traffic. Specifically, the paper proposes a new model called BiADG, which combines BiLSTM, Attention, and DGCNN. This model performs three main tasks: (i) building and synthesizing the behavioral profile of IPs in network traffic, (ii) extracting the behavior of IPs, and (iii) classifying IPs. To build the behavioral profile of IPs in network traffic, this study uses a combination of two methods: BiLSTM and Attention (BiA). In this approach, the BiLSTM network is used to synthesize and to extract the behaviors of IPs in the network traffic, while Attention is used to find and highlight the important IP information. After obtaining the behavioral records of IPs, the system proceeds to build a relationship between the IPs to see the correlation between them. To accomplish this task, the study proposes using a graph to construct the relationship between the IPs. Next, to extract the information on the IPs in the behavioral records, the article suggests using DGCNN. Accordingly, the DGCNN aggregates and extracts features and behaviors that demonstrate the relationship between IPs.

(b) The scientific basis of the proposed method.

Based on the analysis description of the operation process of the BiADG model above, the BiADG model is suitable for the APT IP detection task due to the following main reasons:

- Firstly, it is about the suitability of the BiLSTM-Attention combination model for the task of extracting IP features through flow networks in network traffic. In this study, we do not extract the attributes that show the difference of APT IP in Network traffic such as: Long-cycle, low-frequency, and slow-speed, Abnormal protocols and ports, A large difference between the transferred and received data, Abnormal TCP connection, Abnormal data fluctuation…. These attributes are very difficult to extract in practice, because to extract them requires a very large storage system, as well as many different data sources. Therefore, in this article we have sought to extract information, characteristics, and behavior of IP in network traffic through the flow network. We realize that the flow network is a sequence data type. Using deep learning networks such as Long Short Term Memory (LSTM), BiLSTM will be well suited to extract information based on flow networks. In this paper, we proposed BiLSTM because the strength of BiLSTM is the ability to learn and remember in 2 dimensions, so it can extract distant attributes and features. Next, after the IP information is extracted through the flow network, if only using techniques such as Inference or Mean to calculate, much important IP information will be lost. Therefore, we proposed to use the Attention network. The Attention network with other components will help highlight important and meaningful information instead of just extracting it by averaging like traditional approaches. Thus, combining the two advantages of BiLSTM and Attention networks, the proposed BiADG model has successfully and most completely built the behavioral profile of each IP in the Traffic Network helping monitoring systems to identify new campaigns of APT attacks.
- Secondly, it is about the suitability of the DGCNN model for the task of synthetic feature extraction and IP feature extraction in graph form. We see that each IP will have a different number of flows: Some IPs have tens of thousands of flows, some IPs have only a few flows. Using traditional machine learning techniques for IP classification will not yield good results. Therefore, we need another method to standardize IPs as well as represent information and relationships between them. For this reason, we proposed to use graph techniques to construct and represent relationships between IPs. To synthesize and extract IP information in graph form, there are some traditional approaches such as Grap2vec or Graph Convolutional Networks. According to our survey of data, each IP is built into a graph with vertices as IP and edges as flow characteristics, then the graph of IP will be non-Euclidean data. So if we embed graphs of this type with some traditional graph embedding techniques, it will not be very effective. Non-Euclidean is a type of data that cannot be defined in space, so to fully exploit the information of this type of data, it is necessary to use more advanced graph networks. DGCNN is one of the typical representatives. In this paper, we proposed to use DGCNN to extract features of APT IP.

### Contribution of this paper

The contribution of this research is threefold as below.

- This study introduces the BiADG model for APT attack detection. BiADG is a new model that has not been used before. The three main components in this model are flexibly combined together to not only improve the accuracy of APT attack detection but also reduce the false alarm rate of the system. The experimental results in different scenarios show that this new approach is better than many current methods.
- A proposed method for optimizing the synthesis process and building an IP behavior profile using BiA. In this new method, the statistical features of the flow network are not directly fed into the BiLSTM. Instead, they are divided into several groups according to their different functions. After that, each of these groups is input to the BiLSTM network for training and presenting the behavior of the flow. In this approach, more important and meaningful properties can be extracted in comparison with just putting all the information of the flow directly into the deep network. After that, when the data is processed through BiLSTM, it is further processed through an Attention network to highlight important and meaningful information. The experimental results in section “The results” of the article demonstrate this advancement.
- Proposal of a method for extracting and classifying APT IPs through the behavior profile of IPs. Specifically, in this study, we proposed the DGCNN model to extract the most complete information on abnormal behaviors of APT IPs based on the relationships of IPs in the system.

The rest of the paper is organized as follows: In section “Related works” *“Related Work”*, we study and examine some previous studies for the task of APT attack detection. The contents related to the proposed method are analyzed and presented in section “The proposed method”. The experimental results and evaluations of the effectiveness of the proposed method are presented in section “Experimental results” *“Experiments and evaluations”*. Finally, conclusions and future development directions are presented in section “Conclusions” *“Conclusion and development directions”* of the paper.

## Related works

### Traditional approaches

Xue et al.^21^ proposed a method for detecting APT attacks based on DNS logs. In their study, the authors used several attributes extracted from the DNS log such as Domain Length, Number of Visits to the Domain, Access Period, Digital Feature, Packet Length Characteristics, Domain Name Request and Response Interval, Domain Access Interval, etc. However, we believe that detecting APT attacks based on such attributes is challenging because of the insufficient data and time to obtain abnormal behavior. Muhammad et al.^22^ proposed a method to counter APT attacks in mobile fog computing security using the Double Q-learning algorithm. In the study^23^, Fan Shen proposed the SR2APT model using a graph convolutional network (GCN). The experimental results in the paper showed that the SR2APT model achieved 94% effectiveness compared to other machine learning and deep learning algorithms such as Convolutional Neural Networks (CNN) and Long Short Term Memory networks (LSTM). However, we believe that building a graph like this study is difficult because it requires specific data. Article^24^ proposed using static analysis Bayesian belief network, dynamic analysis Bayesian belief network, and Bayesian belief network to analyze and recognize APT. In the experimental part, the research achieved a relatively high rate with an accuracy rate of 92.62% and an error rate of only 0.0538%. Jaafer et al.^25^ proposed a method for detecting APT attacks based on each stage of the attack campaign, such as reconnaissance, initial compromise, lateral movement, and data exfiltration activities using machine learning algorithms such as random forest (RF), decision trees, K-nearest neighbor, etc. The experimental results showed that the authors’ proposal achieved high effectiveness on 12 groups of flow attributes at 99.89%. We believe that detecting APT attacks at each stage of development is one of the good approaches. However, when evaluating experimental results, such results are not feasible when detecting APT attacks. Their experimental dataset may have been well-balanced and evenly distributed. Ghafir et al.^26^ developed a MAPT model for APT detection using machine learning algorithms based on 3 main stages including: threat detection, alert correlation, and attack prediction. Experiments were conducted using different machine learning algorithms, such as Decision Tree, K-nearest neighbors, Support Vector Machine (SVM), Ensemble, and the network traffic data-set collected in the university. It is shown that the MAPT system has the detection accuracy of 84.8%. Studies^27,28^ have presented some new research directions and approaches for detecting APT attacks. However, implementing and configuring these approaches successfully in practice requires complex and difficult computations. The system is cumbersome and requires high configuration.

### APT attack detection approach based on behavior profiling analysis

In the study^12^, the authors proposed a method for detecting APT attacks based on the behavior profiles of domains and IPs and a supervised machine learning algorithm. The authors attempted to collect abnormal behaviors of APT based on both domain and IP, and then built and combined features of both Domain and IP into an event sequence for detection. However, in that study, it was found that extracting features of domains and IPs was very difficult because it required collecting and monitoring data for a long time. Sometimes, attributes may be meaningful but cannot be obtained in practice.

Their experiments showed that the CNN-LSTM model was better than some other methods using some performance metrics. CNN-LSTM model is also the solution for APT attack detection based on network traffic^17^. In the paper, the authors proposed a CNN model for extracting flow attributes and then used an LSTM network to classify the flows. To classify APT IP addresses, the authors used a traditional voting method. We believe that this method is not effective because, in reality, an APT IP address has a very large difference between begin flows and malicious flows. Therefore, relying on this technique is ineffective and overlooks many APT IP addresses. In fact, in this paper, when we conducted experiments, the results of^17^ were lower than our method. Cho et al.^16^ proposed an APT attack detection method using an associative deep learning algorithm. In their research, a deep learning model based on the combination of BiLSTM and GCN, called BiLSTM-GCN, is developed. They also applied some machine learning models, such as Multi-Layer Perceptron (MLP) and GCN to monitor and detect APT attacks based on network traffic data. Experimental results showed that BiLSTM-GCN is better than other machine learning models in terms of all performance metrics. This approach is the first to use a GCN network to extract attributes of APT IP. However, the grouping of flows by IP addresses is not effective, leading to the inefficiency of this method for the task of detecting APT IP. Cho et al.^11^ proposed a method to detect APT attacks based on network traffic using behavioral profiling techniques. In their research, the authors proposed a method to analyze network traffic into different components such as DNS log, TLS, HTTP, then, the correlation between these components is calculated. Experiments are conducted with different machine learning algorithms, such as RF, SVM, and MLP, to evaluate the attack behavioral profiles. The results show that the RF algorithm has better performance than other algorithms. We believe that this approach brings good effectiveness as it has calculated the correlation of APT IP through various components in network traffic. However, this method requires many different computation techniques, leading to a long processing time and difficulty in practical application. In addition, combining IP and domain also leads to many cases that need to be calculated and processed, which can easily confuse. In their study^47^, Cho et al. proposed a method to detect APT IP based on the analysis of anomalous connections. However, their approach is still relatively simple, which is not able to confirm the types of APT IPs. In other words, that method can only detect anomalous connections based on the flow. In addition, to detect APT malicious code on a user’s machine, the authors in^48^ were the first to propose the use of Grap2vec deep learning graph network in combination with BiLSTM and LSTM network to extract and classify APT malwares. Following the same track, Do et al.^49^ presented a solution to detect APT malware based on the Graph Isomorphism Network (GIN) network. This is a relatively new approach to APT malware detection. In their experiments, the authors compared the efficiency of 3 deep learning graph networks, i.e. GCN, DGCNN and GIN, in APT malware detection. Experimental results show that the GIN network has a better performance than the others. However, during the experiments, they did not implement some techniques to fully utilize the characteristics of the APT malwares. In order to tackle this problem, Cho et al.^50^ proposed an intelligent computational method based on the combining graph embedding and Attention model. Experimental results show that the study in^50^ was more effective than the studies in^48,49^. Overall, the articles^47–50^ have been successful in building behavioral profiles of APT attacks based on graph networks. However, these articles have not been effective in synthesizing and representing the relationship between APT IPs, leading to lower experimental results. To address this issue, the authors of the study^51^ proposed combining deep graph networks and inference networks. The inference network is responsible for representing the relationship between flows according to APT IPs. In the experimental section, the authors compared their research approach with many other results and showed that the study^51^ yielded the best results, with an accuracy rate of over 84% for accurately detecting APT IPs and over 86% for normal IPs. However, we found that the approach using the inference network had a problem in that it represented all the relationships between flows but did not clarify the important information and significance of the features in the flow network, leading to lower classification results. In this paper, we propose a method to improve the research approach in the study^51^. The experimental results in our paper yielded better results than the study^51^ by about 4% across all metrics. This is the best result to date based on this experimental dataset.

## The proposed method

### The model architecture

**Fig. 1:**
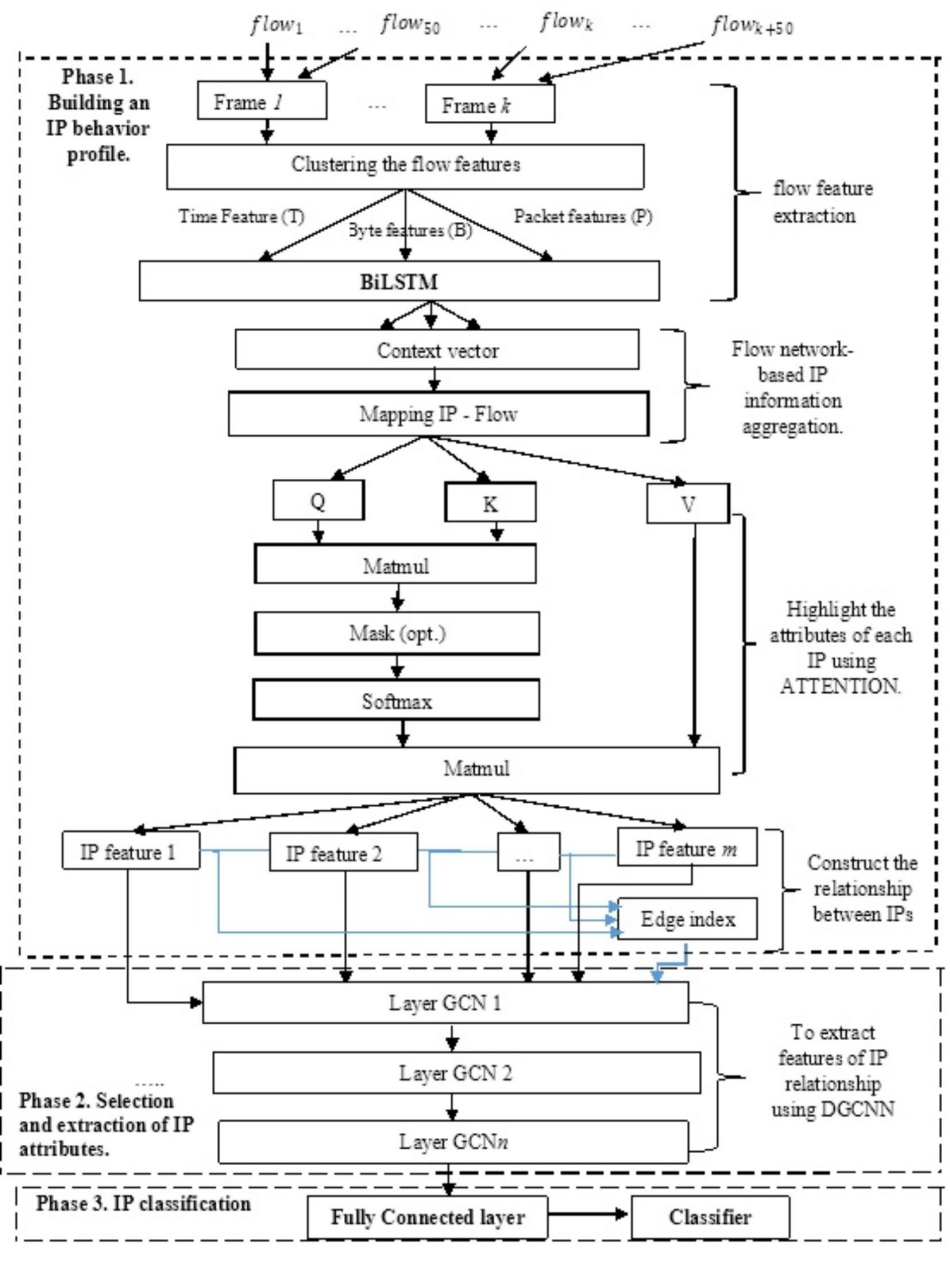
The model of detecting APT malware based on workstations and analyzing the process profile using deep learning.

Figure 1 below illustrates the working principle of the BiADG model for APT attack detection. From Fig. 1, it can be seen that the operating principle of the BiADG model includes following phases with different phases:

- **Phase 1—Building the behavior profile of IP in network traffic**: To build the behavior profile of an IP address, we propose the following steps. Step 1: The flow features are extracted and synthesized from the network traffic using a BiLSTM network in the flow feature extraction phase. Step 2: The IP information is aggregated based on the flow network to derive the information of each IP in the subsequent phase. The flows are first calculated and processed through the BiLSTM network, those that have the same IP address are then grouped. This process helps build the most complete information of the IP through the flow network, based on which the IP classification process can be implemented. Step 3. To highlight important behaviors of the IP using Attention network: Based on the anomalous behavior of the IPs extracted from the flow through the BiLSTM network implemented in step 2, the important features and behaviors of the IPs are then searched and highlighted. Attention networks are used in this step.
- **Phase 2—Extracting IP behavior through behavior profiles**: There are two steps for IP feature extraction and selection based on flow networks as follows: Step (1) To construct the relationship between IPs: In this step, the relationship between IPs are presented through the flow networks. Specifically, IPs that exchange information with each other are used to build graphs, in which vertices are IPs and edges are the features of the flow network; Step (2) To extract the features of the IP relationship: in this step, DGCNN is used.
- **Phase 3—IP classification**: In this phase, the IPs are classified as APT IPs or normal IPs.the.

### Building IP behavior profiles

#### Flow feature extraction

As illustrated in Fig. 1, the flow feature extraction and aggregation process includes multiple steps, which are: flow analysis from network traffic; flow feature clustering; flow information extraction using BiLSTM. These steps are detailed as follows.

- Flow feature clustering.

The CICFlowMeter^29^ tool is utilized to derive flow networks from network traffic, with the tool being responsible for both analyzing the network traffic to create flow networks, as well as extracting statistical features from those flow networks. In the study^16^, 75 attributes of flow in network traffic were listed. Specifically, the CICFlowMeter tool extracted 75 features of the flow network which were then clustered into different groups. The clustering process was based on grouping features with similar characteristics, purposes, and formats together. Consequently, the 75 features were categorized into three main groups: byte features (B), time features (T), and packet features (P). Group B consists of attributes from the first to the 28th, group P includes attributes from 29 to 64, and Group T comprises attributes from 65 until the end.

(b) Summarizing and extracting flow attributes using BiLSTM.

In the research presented in^30^, the BiLSTM architecture was proposed, comprising both forward and backward LSTM components. This design not only enables the model to retain the long-term memory capability of LSTMs but also provides the capacity to process and memorize bidirectional information. The two layers of LSTM generate two corresponding hidden states, *h*^*f*^_*i*_ from forward LSTM and *h*^*b*^_*i*_ from backward LSTM. *h*^*f*^_*i*_ integrates forward information, while *h*^*b*^_*i*_ integrates backward information. The final state *h*_*i*_ is defined by mapping the two states using a connection formula as follows.

1 $${h_i}=h_{i}^{f}\left\| {h_{i}^{b}} \right.$$

Where: *h*_*i*_ is the state at time *i* (containing the information from 2 directions); || is the connection operation.

The novel model introduced in this study can acquire an extra dimension of information, as evidenced by the architecture of the BiLSTM network and formula (1). This helps greatly improve the memorability during the feature extraction process, then helps extract important features and behaviors. In this paper, the two consecutive deep network layers of BiLSTM are used to extract flow information from 3 feature groups (T), (P) and (B) mentioned above. First, the flows are divided into clusters, each of which consists of 50 flows, to feed into the first BiLSTM layer consisting of 128 hidden states. Second, the output of the first BiLSTM layer is fed into another BiLSTM layer consisting of 256 hidden states aiming at synthesizing deeper properties of the flow. This approach of using the BiLSTM network helps reconstruct and synthesize important information of the flow. The outputs of a 2-layer BiLSTM network are three context vectors representing three groups of features (T), (B), (P).

#### IP information aggregation based on flow network

Three feature groups, (T), (B), (P), are extracted from the two-layer BiLSTM network. The context vector corresponding to the attribute group (T) contains aggregated information about the time of the cluster of 50 flows, while that of the group (B) has information about bytes, while the context vector of group (P) provides information about the packets. At the end of the flow network information aggregation process, these 3 context vectors are concatenated together into a single context vector carrying the aggregate information of the flows. Next, the flows are grouped based on IP addresses. Specifically, each IP corresponds to a different and inconsistent number of flows. 50 frames for each IP is selected. IPs with less than 50 frames are filled with flows having a value of 0. IPs with more than 50 frames are cut off to 50 frames randomly. From this point, each 50-frame IP is analyzed and evaluated to find the most important and meaningful attributes. This process will be further discussed in section “IP feature extraction based on behavior profile”.

#### IP feature extraction using attention network

Attention is one of the most interesting models in deep learning^31^. Dzmitry et al.^32^ were the first to propose the use of Attention network for the machine translation systems. Jiachen^33^ applied Attention to implement text classification. Colin Raffel^34^ used the Attention model to solve the problems with strings with medium or large length. In this paper, the Attention network is adopted to extract and highlight important information about IP based on flow. To be more specific, the Attention network is utilized to process each IP that contains 50 context vectors for 50 frames to extract and combine its characteristics, allowing the model to learn and identify the frames with the most significance. The proposed Attention network architecture is illustrated in Fig. 1. In this approach, the Attention network consists of 4 main layers with different tasks. The detailed processing procedure on IP frames in each layer of the Attention network is presented in four layers as follows.

- Layer 1: *Q*,* K*,* V* matrices are extracted from context vectors. Matrices *Q* and *K* are used to distribute scores among frames of each IP. Matrix *V* is used to aggregate scores. First, the Attention network uses the input block of IPs with 50 context frames to calculate the *Q* and *K* matrices. Second, the model uses the *V* matrix to aggregate the scores and output a final Attention vector. The general formula of blocks *Q*,* K*,* V* is presented by the following formula.
  2 $$Q=X.{W_Q},\;\;K=X.{W_K},\;\;V=X.{W_V}$$
  Where, *W*_*Q*_, *W*_*K*_ and *W*_*V*_ are weight matrices that need to be trained.
- Layer 2: to calculate scores between frames. This layer includes two main steps. First, the two matrices *Q*, *K* found in Layer 1 are put through the dot-product operator to find the weighted relationship between each pair of frames in the IP. Second, the resulting matrix is divide by ***d***^***k***^^35^ to avoid overflow if the power is too big. The score is calculated using following formula^36^:
  3 $$score=\frac{{Q.{K^T}}}{{\sqrt {{d^k}} }}$$
  Where, $\:{d\:}^{k}$ is the number of dimensions of vector *K.*
- Layer 3: score normalization. The scores from two matrices Q, *K* are normalized. Here, the Softmax function is used to convert the score to a probability distribution whose magnitude represents the attention level of the two frames. The larger the weight, the more attention and the more effect to each other of the two frames. The formula for normalizing the score is as follows.
  4 $$\:Pscore\:=\:softmax\:\left(\frac{Q{K}^{T}}{\sqrt{{d}^{k}}}\right)$$
- Layer 4: Attention vector extraction. The P scores of the frame pairs obtained at Layer 3 are aggregated to produce an Attention vector that presents the score information of those frame pairs. The dot-product between the score matrix and the V-matrix from layer 1 is performed to find the Attention vector for each IP after synthesizing all the Attention information from the frames corresponding to that IP. Formula (5) below summarizes the process to extract the Attention vector.
  5 $$\:Attention(Q,K,V)\:=softmax\:\left(\frac{Q{K}^{T}}{\sqrt{{d}^{k}}}\right)V$$

### IP feature extraction based on behavior profile

In this research, the IP feature extraction is performed in two different approaches. In the first approach, each IP with 50 frames is analyzed using the Attention network. In the second approach, the features are extracted based on the relationship between IPs. Each IP has various external relationships with other IPs. Only relationships between IPs through the flow network are built and exploited. DGCNN is used in this stage. More details of these two feature extraction techniques are presented in sections “IP relation construction” and “IP relation feature extraction based on DGCNN” of this paper.

####  IP relation construction

It can be seen that each IP has relationships with other IPs through the linking and exchanging information processes. The behaviors of these relationships can be considered as an important source of information and may contribute a significant role to the assessment if an IP is normal or malicious. From this point of view, features extracted from the relationship between IPs can be useful for APT attack detection. The principle of building the relationship between IPs is as follows: if 2 IPs communicate with each other, they are marked as related, then, an edge in the graph connecting these 2 vertices corresponding to these 2 IPs is established. After traversing all the communications among all the IP pairs, a complete graph with the vertices corresponding to the IPs and the edges representing the communications within pairs of IP vertices is constructed.

#### IP relation feature extraction based on DGCNN

The DGCNN model was first proposed by Zhang et al. in 2018^37^. In this model, the GCN layer is similar to the conventional GCN algorithm. The difference between those two is a change in the attribute propagation process. Equation (6) below shows how this layer in DGCNN works.

6 $$Z^{{(i + 1)}} = \sigma \left( {\tilde{D}^{{ - 1}} \tilde{A}Z^{{(i)}} W^{{(i)}} } \right)$$

Where:

*Z*^*i+1*^: output of layer i + 1;

*σ*: activation function;

*D*: degree matrix;

*A*: adjacency matrix;

*I*: unit matrix with the same size as A;

$$\tilde{A} = A + I ;$$

*W*: weight matrix.

**Fig. 2:**
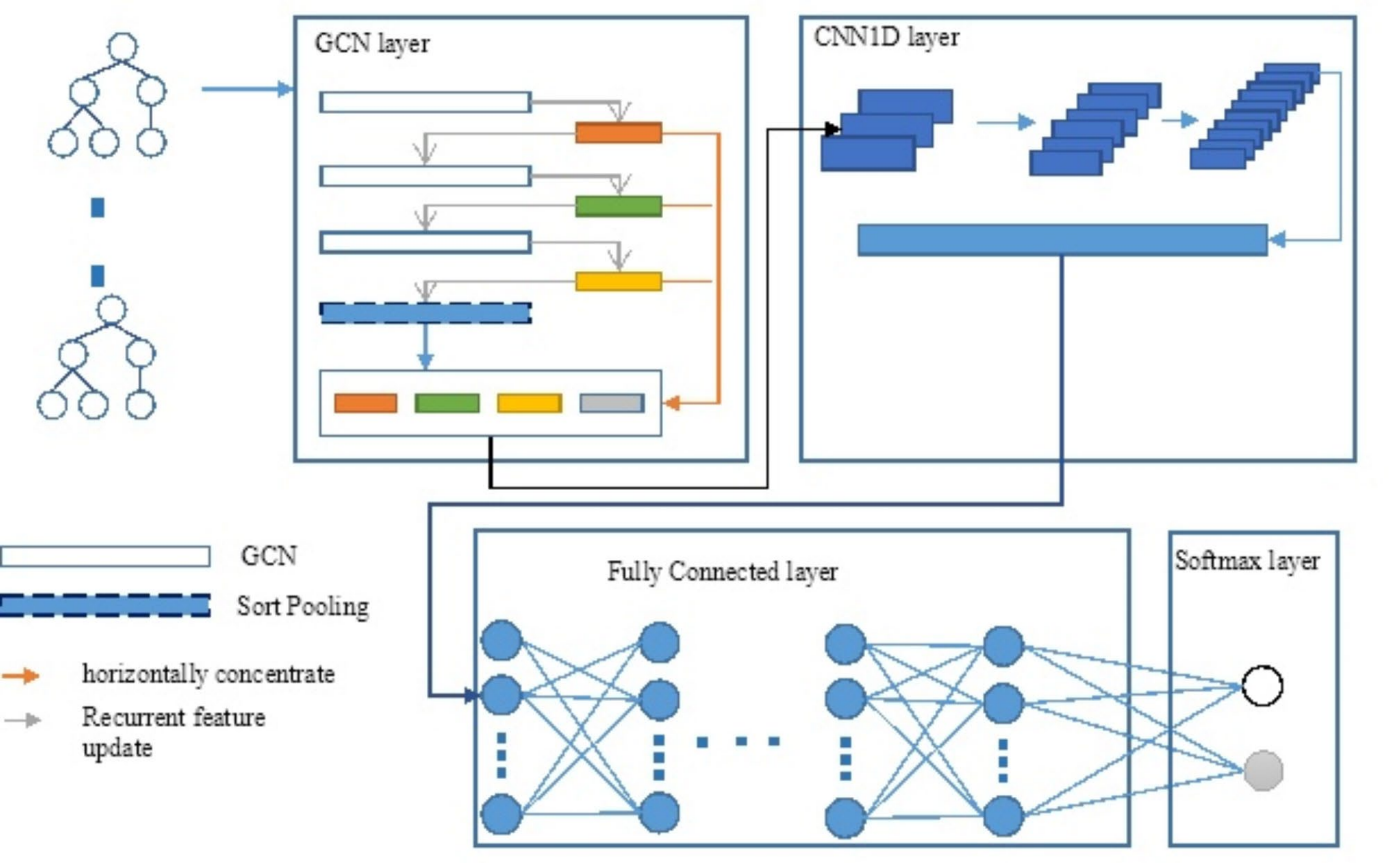
Architecture of DGCNN for information extraction.

In the studies^38–42^, some practical applications of DGCNN were listed. In this paper, we propose using DGCNN to extract IP attributes based on their behavioral profiles. Specifically, the input of the DGCNN is the graph network of IPs built in (a). The DGCNN treats IPs as the vertices of the graph. Figure 2 illustrates the structure of the DGCNN model used for extracting relational features.

The diagram highlights that the DGCNN model comprises three primary components for processing and analyzing the data. GCN layer and CNN1D layer are used for data processing, and Fully Connected and Softmax layers are used to classify data. Details of these layers are presented in section “IP classification”. Followings are the procedure to process and extract IP behaviors through the DGCNN.

- GCN layer: In the DGCNN model, the GCN layer outputs a set of features for each vertex in the graph, which are then combined horizontally. This process aims at presenting the basic structure of the graph in more detail through subgroups, which is different from using only features from the last layer. Besides, GCN layers of DGCNN apply Sort-Pooling, which is responsible for not only ensuring the uniformity of the size, but also specifying the order of the outputs of the GCN layers.
- CNN1D layers: the CNN1D layer is used to cope with the output of the GCN layer, which is a set of feature nodes arranged in a descending order.

### IP classification

Figure 2 presents the classification of IPs into APT or normal based on two layers, namely the Fully Connected Layer and Softmax Layer. The Fully Connected Layer, which functions like the MLP network, learns the attributes processed through the DGGCN layers. For more information on the Fully Connected Layer’s operation, refer to^43^. On the other hand, the Softmax Layer calculates the output label probability using the adopted Softmax function^36^.

## Experimental results

### The data

Table 1 shows the statistical information of experiment data that are collected and used in this paper.

**Table 1:** Details of the experimental data.

*N* ^o^	Type	Total	Malicious	Normal
1	Flows	8.543.362	19.025	8.524.337
2	IP	157.126	7.375	149.751

The research study gathered positive experiment data (attack data) from^44^. We have searched and collected data about APT attacks on network traffic in Pcap file format from known APT attack campaigns. Specifically: The positive experiment data (attack data) was collected from 29 Network Traffic files in the Malware Capture CTU-13 data set which contains 7 types of malwares from the APT attacks, including: Andromeda, Colbalt, Cridex, Dridex, Emotet, and Gh0stRAT, Mustang Panda. These are malwares samples proven to be APT attacks. Meanwhile, the negative experiment data (normal data) was obtained from the E-Government server of Soc Trang province^45^. During the experiment, the data set is divided into two subsets, which are the training subset accounting for 80% and testing subset accounting for remaining 20%.

### Performance evaluation metrics

#### The metrics

Four main metrics are used to evaluate the model during the experiments, including: Accuracy, Precision, Recall, F1-score. The general formula for these four measures is as follows.

8 $$\:Accuracy=\frac{TP+TN}{TP+TN+FP+FN}\times\:100\%$$

9 $$\Pr ecision=\frac{{TP}}{{TP+FP}} \times 100\%$$

10 $$\operatorname{Re} call=\frac{{TP}}{{TP+FN}} \times 100\%$$

11 $$\:F1=\frac{2\times\:Precision\times\:Recall}{Precision+Recall}$$

Where:

- **Accuracy**: is the number of correctly predicted samples out of all the samples.
- **Precision**: is the ratio of true positive points to the total number of points classified as positive (*TP* + *FP)*.
- **Recall**: is the ratio of true positive points to the total number of real positive points (*TP* + *FN)*.
- **F1-score**: is the harmonic mean of precision and recall.

#### Evaluation scenarios

Some experimental scenarios are conducted as follows:

- Question 1: Detecting APT attacks using the BiADG model. In this scenario, the parameters of the BiADG model will be modified to find the best model.
- Question 2: Comparing the BiADG model with other models. In this scenario, the article replaces each component of the BiADG model with different algorithms. Specifically, replace the BiLSTM network with other deep learning networks such as CNN, RNN, and LSTM. Replace Attention with Mean and Inference networks. Finally, replace DGCNN with the GCN deep graph network or some other machine learning and deep learning algorithms such as MLP and RF.
- Question 3: How does the BiADG model outperform other models? The experiments are conducted to compare the proposed model with some other approaches in detecting APT attacks, including: BiLSTM-GCN^16^; CNN-LSTM^17^; MLP-Inference- GCN (MIG)^51^.

### Hyperparameter configuration

Table 2 below lists the parameters used for the experiment BiADG Frameworks in the paper.

**Table 2:** Selecting and configuring the hyperparameters of the BiADG Framework.

Component	Hyperparameter	Value
BiLSTM—Layer 1	Hidden Size	128
BiLSTM—Layer 2	Hidden Size	256
DGCNN	Hidden Size	256
	Number of Graph layers	8
	Learning Rate	1e-4
Attention	Hidden Size	256
Classifier	Batch size	32
	Activation	Softmax
	Learning Rate	1e-4
	Loss Function	Cross-Entropy

### The results

#### Experiments for question 1

The following Table 3 shows some experimental results of Scenario 1 in the article.

**Table 3:** Experimental results for APT attack detection based on BiADG with different parameter setups.

Data-set usage	Bi-LSTM + Attention + DGCNN			IP classification performance			
	Bi-LSTM nodes	Attention nodes	DGCNN nodes	Acc	Pre	Rec	F1
75 features	128–128	128	256	0.98	0.82	0.84	0.83
	128–256	256	256	0.99	0.84	0.88	0.86
	256–256	256	512	0.97	0.69	0.81	0.75
3 feature groups proposed in this study	128–128	128	256	0.98	0.92	0.83	0.87
	128–256	256	256	0.99	0.92	0.91	0.91
	256–256	256	512	0.99	0.93	0.86	0.89

**Fig. 3:**
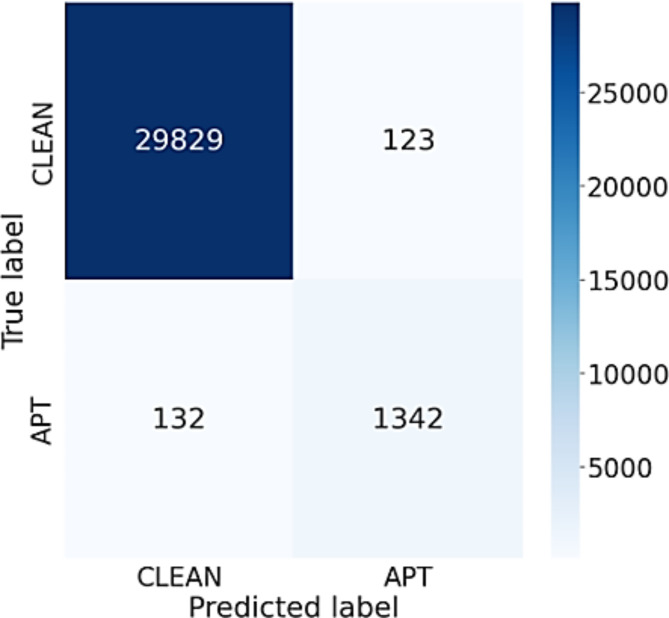
Confusion matrix of BiADG model using feature clustering approach.

During the experiment, a different number of nodes are set for 2 layers in the BiLSTM network, 2 layers of Attention and 2 layers of DGCNN. The results show that most of the evaluation metrics of the proposed model using the feature clustering approach are better than using the original 75 features. The resulting differences vary from 1 to 23%, which is significant. In particular, the proposed model has better ability to accurately detect APT IPs using the recommended three feature groups compared to using traditional 75 features. The performance difference is from 2 to 10%. Regarding normal IP detection, the performance difference is from 10 to 24%. The reason for this advancement is that the division of the original 75 attributes into 3 groups, i.e. time (T), byte (B) and package (P) features, helps enable the model to focus on extracting and synthesizing better information within each particular group. It is found that the features from the three groups (T), (B) and (P) often have little relation as well as influence on each other, which results in better classification due to less correlation compared to directly aggregating information from the original 75 features. Furthermore, distinct configurations of the model’s parameters can result in varying outcomes during experimentation. This change varies across all measures. For Accuracy and precision measures, the results change insignificantly, which is only about 1%. As for the IP APT classification results, there is a big difference between the models. This difference ranges from 3 to 8%. With the parameter configuration of [128–256; 256; 256], the model achieved the highest accuracy in all performance measures. The confusion matrix of this model is displayed in Fig. 3, which indicates that the model accurately predicted APT IPs and normal IPs, as shown in the figure. Specifically, this model correctly predicted 1342 APT IPs, while only miss-classified 132 APT IPs. Besides, the model worked better in classifying normal IPs, when only falsely classified 123 IPs out of a total of 29,952 normal IPs.

Discussion: experimental results show that with an unbalanced data set as shown in Table 1, the BiADG model with the flexible combination of BiLSTM, Attention and DGCNN has succeeded in synthesizing and extracting information from the data which helps improve the classification performance.

####  Experiments for question 2

The issue at hand is whether alternative networks can be utilized in place of the deep learning networks employed in this model. Some other questions can be how to clarify in the proposed model, or which network in the proposed model plays the most important factor and mostly determines the accuracy of the whole system. To answer these questions, three different experiment scenarios (a, b, c) below are implemented.

- To evaluate the efficiency of BiLSTM.

To evaluate the efficiency of the BiLSTM network in the proposed model, some other network structures such as CNN, LSTM, and RNN are used. These replacements are all deep learning networks, which are well known for their efficiency at aggregating and extracting information. The experimental results of various model configurations are presented in Table 4. Various parameter setups are investigated for each network, and their best results are recorded.

**Table 4:** Experimental results when replacing BiLSTM by different networks.

Parameter		Evaluation metric			
Network	Data usage	Acc	Pre	Rec	F1
CNN	75 features	0.97	0.70	0.62	0.65
	3 feature groups as proposed in the study	0.98	0.79	0.71	0.73
LSTM	75 features	0.98	0.86	0.79	0.83
	3 feature groups as proposed in the study	0.98	0.82	0.85	0.84
RNN	75 features	0.97	0.90	0.73	0.8
	3 feature groups as proposed in the study	0.97	0.84	0.76	0.79

**Fig. 4:**
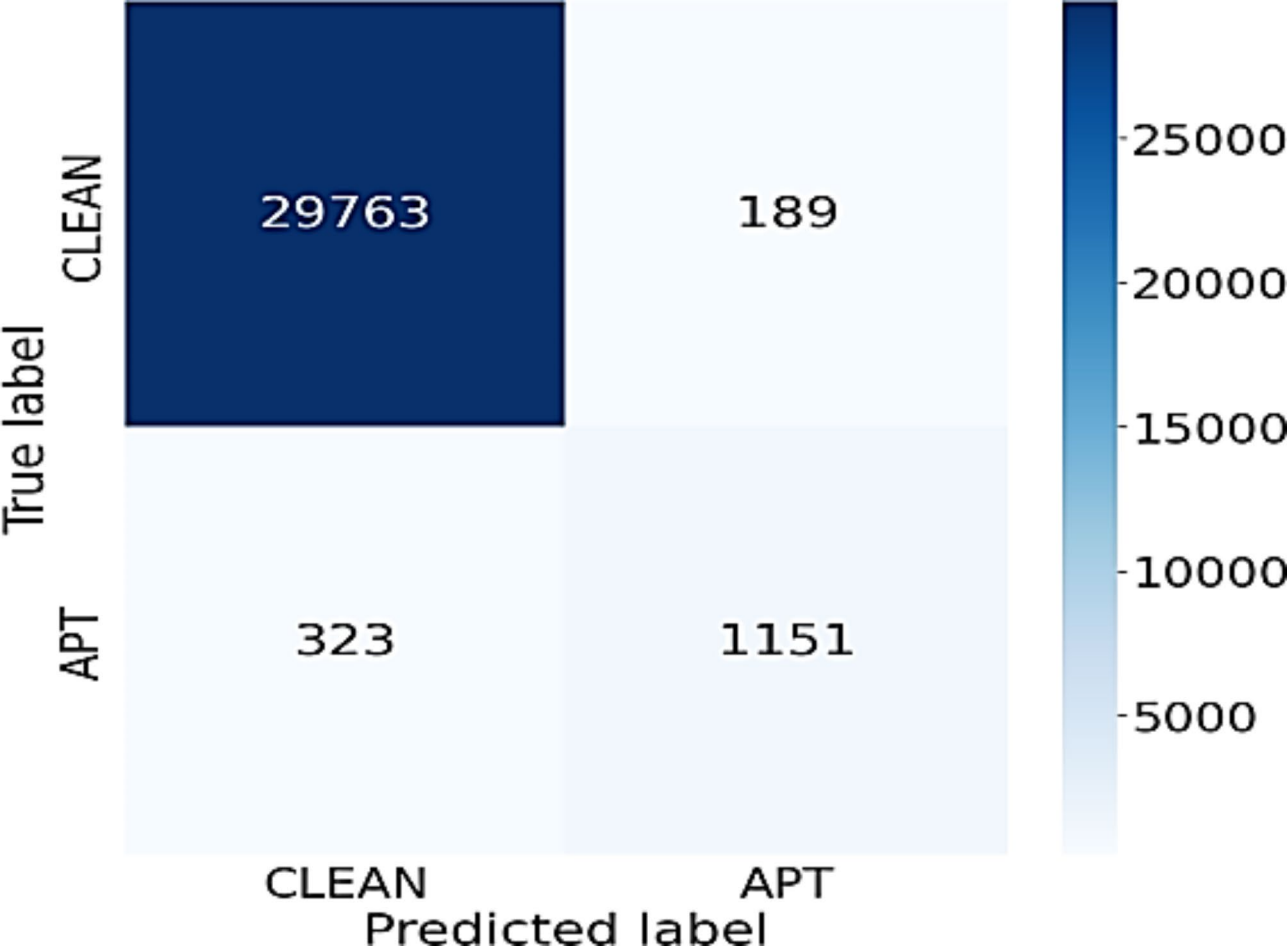
Confusion matrix of LSTM network using 3 feature group approach.

The experimental results in Table 4 show that when the BiLSTM network is replaced by some other networks, the efficiency of each different model is also different. Besides, using a 75-feature setup yields lower efficiency than using the 3 feature groups approach as suggested in this study. Specifically, performance measures of CNN using 3 feature groups are 98%, 79%, 71%, 73% on Accuracy, Precision, Recall and F1-Score, respectively. These results are better than using the original 75 features. The differences are 1%, 9%, 9% and 8% in Accuracy, Precision, Recall and F1-Score, respectively. Similarly, the LSTM network is also efficient for classifying normal data and APT data. When using the approach according to three feature groups, the LSTM network also gives better results than the original 75 features. Additionally, the LSTM network works better than the CNN on all performance measures. The results for RNN are similar to that of CNN. When comparing these 3 networks, the LSTM network yields higher results than the other 2 networks. Figure 4 below shows the confusion matrix of the LSTM network using the 3 feature group approach. From Fig. 4, it can be seen that the LSTM network is efficient for detecting APT attacks. Specifically, this network has correctly detected up to 1151 APT IPs with a rate of 79.3%. This model also correctly predicted 29,763 normal IPs, while misclassified only 189 IPs. The results show that the LSTM model has a higher efficiency for classifying the normal IPs than detecting the APT IPs.

Observing the outcomes presented in Tables 3 and 4, it is evident that the significance of BiLSTM in the suggested model cannot be overstated. When replacing the BiLSTM network with other networks such as CNN, LSTM, and RNN, the efficiency of APT attack detection system downgrades significantly. This is because the BiLSTM network with a 2-way memorization process can extract and aggregate more important properties of the flow, thereby helping to build the most complete IP information, making the prediction process easier. When comparing the data presented in Figs. 3 and 4, it becomes apparent that the BiLSTM network provides significantly better predictive results than the LSTM network. Specifically, the miss classification score for APT IPs of the BiLSTM model is 3 times smaller than the LSTM model, while the false alarm rate for the normal IPs of the BiLSTM is just one half of that of the LSTM network.

(b) To evaluate the efficiency of Attention network.

The purpose of the Attention network in this study is to aggregate and highlight important information of the flow in the IP instead of equally treating all features. In this scenario, the Attention network is replaced by two other networks, which are Inference^46^ and Mean. Table 5 below presents the experimental results of these two models.

**Table 5:** Experimental results when replacing attention network by two other networks.

Parameter		Evaluation metrics			
Networks	Data usage	Acc	Pre	Rec	F1
Mean	75 features	0.97	0.82	0.76	0.78
	3 feature groups as proposed in the study	0.98	0.84	0.78	0.79
Inference	75 features	0.98	0.83	0.84	0.8
	3 feature groups as proposed in the study	0.99	0.85	0.85	0.85

The experimental findings outlined in Table 5 demonstrate that the 3-feature-group approach results in improved performance for both the Inference and Mean networks. However, the Inference network outperforms the Mean network overall. These results suggest that using the Mean network may lead to a loss of significant information related to flow behaviors. In contrast, the Inference network assigns greater weight to flows with unique or unusual values, which are critical signatures in identifying APT attacks. For instance, if an IP has multiple flows and only one of them is abnormal, the Mean network considers all flows to be equally important, thereby obscuring the anomalous flow and reducing the efficiency of synthesizing anomalous IP behaviors from the flow network. On the other hand, by weighting the flows based on their significance and role, the Inference network highlights the characteristics of flows with anomalous values, allowing for complete and diverse extraction and aggregation of information from IPs.

**Fig. 5:**
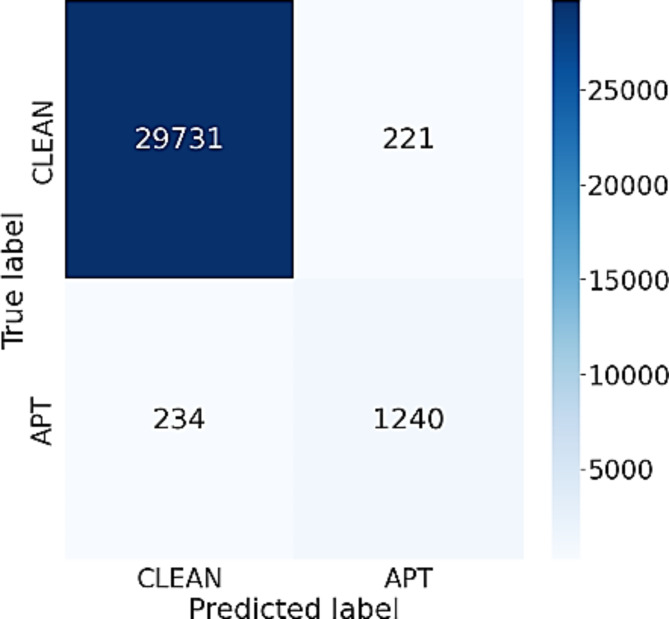
Confusion matrix of Inference network using 3 feature group approach.

Figure 5 illustrates the confusion matrix of the Inference network using the 3-feature-group approach. The data presented in Fig. 5 indicates that the Inference network has delivered a commendable classification accuracy for both normal IPs and APT IPs. More specifically, out of 1474 APT IPs, the Inference network correctly identified 1240 IPs, and for normal IPs, only 221 IPs were falsely identified out of a total of 29,952 IPs. These findings suggest that the Inference network effectively amalgamates crucial characteristics of the network flow, leading to a significant improvement in the detection accuracy of APT IPs.

It can be seen from Tables 3 and 5 that the model using Attention network has yielded better performance than both Inference and Mean networks. Mean network aggregates features by evenly averaging all the values of the flows. This may lead to the loss of important and significant properties. As a result, the APT detection results of the Mean network are not very good. Inference network is better than Mean network when it assigns more weight to flows having exceptional or unusual values. However, this weighting process greatly depends on a composite coefficient β, so the value and the importance of each feature also depend on this coefficient. In practice, the choice of coefficient β is very difficult, so the effectiveness of the Inference network can be limited. Comparing the results of the confusion matrices presented in Figs. 3 and 5, it can be seen that the Attention network can automatically learn the weights for each frame through 3 coefficient matrices Q, K and V. This results in its better classification performance than that of both Inference and Mean networks in terms of detecting both APT IPs and normal IPs.

(c) To evaluate the efficiency of DGCNN.

In this scenario, DGCNN is replaced by some other deep learning networks and classification algorithms such as GCN^23^, MLP, RF. Table 6 below shows some experimental results of this scenario.

**Table 6:** Experimental results when replacing DGCNN with some other models.

Parameter		Evaluation metrics			
Model	Data usage	Acc	Pre	Rec	F1
MLP (best model)	75 features	0.98	0.74	0.83	0.78
	3 feature groups as proposed in the study	0.98	0.84	0.87	0.86
RF (best model)	75 features	0.98	0.84	0.8	0.82
	3 feature groups as proposed in the study	0.98	0.82	0.81	0.8
GCN(best model) ^23^	75 features	0.98	0.74	0.83	0.78
	3 feature groups as proposed in the study	0.99	0.84	0.89	0.87

**Fig. 6:**
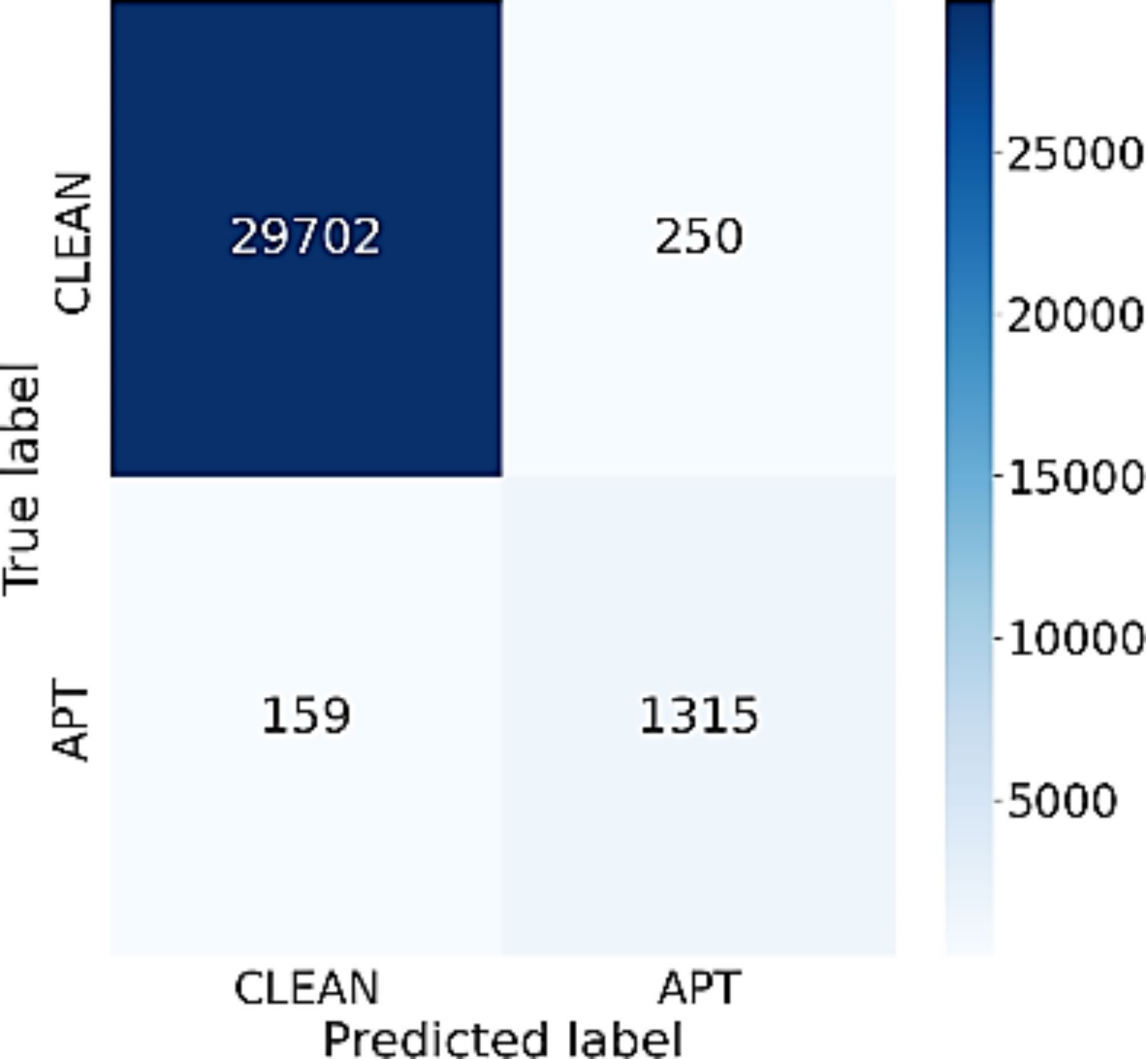
Confusion matrix of GCN model using 3 feature group approach.

In general, the experimental results in Table 6 are quite similar to those of previous scenarios as the experimental models show higher efficiency when applying the 3-feature group approach. GCN is more efficient than both MLP and RF. This implies that the GCN is more effective in extracting IP relationship information. MLP networks work better than the RF algorithm. This makes sense because the MLP network is a deep learning model, so it is able to extract the attributes of the IP, and it can get more behavior information. Figure 6 presents the confusion matrix of the best GCN in Table 6.

By analyzing the outcomes presented in Tables 3 and 6, it is evident that there is a noteworthy dissimilarity in the performance of DGCNN when compared to other substituted networks. Specifically, the classification performance of DGCNN is about 1–8% higher than that of the MLP in all metrics. Especially, the true negative rate for normal IP of DGCNN is 8% higher than that of MLP network. The performance of DGCNN is higher than RF algorithm from 1 to 11% in all metrics. DGCNN’s accurate prediction rates for APT IPs and Normal IPs are approximately 10% higher than those of RF. Finally, the performance of DGCNN is better than GCN by about 2–4% in all metrics. When comparing Figs. 3 and 6, it becomes apparent that the DGCNN outperforms the GCN network in terms of false classification for both APT IPs and normal IPs. The reason for this result might be about the network structure of DGCNN. There are two important components in the DGCNN model that help create better performance than GCN. The first component is the GCN layer inside the DGCNN. The second component is the 1D-CNN. These two components help DGCNN outperform GCN in APT classification.

#### Experiments for question 3

The goal of this scenario is to compare and evaluate the effectiveness of the Bi-ADG model with other studies on the same experimental data set. To prove that, we compared the BiADG model with the MIG^51^, CNN-LSTM^17^, BiLSTM-GCN^16^ models. During the experiment, all parameters of the three models are investigated and only the parameter setup that has the best result for each model is recorded. Specifically, the structure for CNN-LSTM model^17^ is 3CNN-2LSTM. The structure of the BiLSTM-GCN model^31^ is 2BiLSTM-2GCN. The structure for the MIG^51^ model is 2MLP-2GCN and the coefficient β in the Inference network is 0.7. Table 7 below lists the best experimental results of these three models.

**Table 7:** Experimental results for APT attack detection of different approaches.

Parameter	Evaluation metrics			
Models	Acc	Pre	Rec	F1
BiLSTM-GCN^16^	0.97	0.73	0.62	0.67
CNN- LSTM^17^	0.96	0.65	0.40	0.49
MIG^51^	0.99	0.86	0.84	0.85
BiLSTM + Attention + Dropout + contrastive learning^55^	0.99	0.84	0.89	0.87
CIG^56^	0.98	0.78	0.70	0.74

Comparing the results from Tables 3 and 7, it can be seen that the BiADG model proposed in this study outperforms the three other models. The performance of the BiADG model outperforms that of the CNN-LSTM model across all metrics, with improvements ranging from 3 to 51%. The BiADG model achieves double the recall rate (around 51%) and approximately 28% higher precision rate compared to the CNN-LSTM model. These results are mainly attributed to the BiADG model’s ability to leverage both CNN and LSTM, while the CNN-LSTM model can only analyze one flow at a time. Despite the support from the GCN structure, the BiLSTM-GCN model still falls short of the BiADG model in synthesizing the IP features. As a result, the BiLSTM-GCN model is not able to efficiently classify APT IPs and normal IPs. The BiADG model outperforms the BiLSTM-GCN model in all performance aspects. In particular, the recall score of the BiADG is 30% higher than the BiLSTM–GCN model, while the normal IP detection accuracy of the BiADG is 20% higher. Finally, BiADG also has better performance than the MIG^51^ model on all measures. However, this performance difference is not as significant as compared to the other two models. Specifically, APT IP prediction accuracy as well as normal IP prediction accuracy of the BiADG model are about 7% higher than the MIG^51^ model. There are two reasons why the BiADG model provides better results than the BiLSTM-GCN models^16^; CNN-LSTM^17^; MIG^51^. Firstly, BiADG model is suitable for synthesizing APT IP attributes based on flow networks. It is stated in many publications that APT is considered as a malicious attack technique. The danger of APT attack campaigns is based on two factors: the technology and people. Regarding the technology, APT campaigns often apply very advanced techniques and technologies developed by the attackers themselves. Specifically, the types of malicious code designed by the attackers can be very sophisticated, that help them hide and delete traces. Some common ways that APT malwares often exploit to bypass the monitoring of current attack detection systems are using dynamic sleep mode, or using normal processes. Besides, during the connection process, the IPs of the victim machine and the IPs of the attackers often generate a lot of network flows. Therefore, it is necessary to aggregate all flows generated by all IP addresses. After these flows are fully aggregated, there is a basis for recognizing unusual IP behaviors. In this study, BiADG model is based on a deep learning network combined with an ATTENTION network to synthesize and extract attributes of the flows from each IP address. With this approach, the characteristics of the upcoming or ongoing flows are remembered and highlighted. As a result, the BiADG model can overcome the disadvantages of memorization when dealing with a long time series. This is what models like BiLSTM-GCN^16^; CNN-LSTM^17^; MIG^51^ are missing. Secondly, the BiADG model is suitable for the task of extracting and classifying APT IP anomalous behaviors based on the network flows. From the above analysis, it can be seen that each IP may have a different number of flows. Those numbers can be tens, hundreds, or even tens of thousands of flows. However, some IPs may only have one single flow. The challenge here is how to normalize the number of flows in different IPs. It is difficult to standardize the number of flows in each IP because, by nature, it is impossible to predict whether the flows generated before or after the connection are important, or to define the suitable number of the flows in an IP. In order to solve this problem, a graph network becomes the most reasonable solution. Here, each IP is formulated as a graph with IP address are staying at vertices, while edges are flow features. The graph of IP is non-Euclidean data. At this point, some traditional graphing techniques such as Grap2vec or GCN become less efficient. This is because the non-Euclidean data is a data type that cannot be defined in space. In order to fully exploit this data type, it is necessary to use the graph networks, of which DGCNN is one of typical representations. Therefore, the proposal to use DGCNN for APT IP feature extraction is correct and reasonable.

### Discussion

####  Advantages

The experimental results in section “The results” have shown the efficiency of BiADG in detecting APT attacks, even when the experimental data-set is unbalanced. The reasons for those results are two folds. First, the combination of three networks BiLSTM, Attention and DGCNN in the proposed model helps utilize important information from the flow network. Experimental results in scenario 2 shows that when replacing one of these 3 networks with some other commonly used networks, it is not as effective as the BiADG model.

Second, the proposal to extract both the features of the individual IP itself and the features of relationships between IPs has yielded good results. As mentioned above, the idea to divide the flow’s features into 3 different groups and then adopt the BiLSTM network to aggregate the information from each group works well since it enables the extraction of many high-level features of the flow. Experimental results show that most of the models have better performance results when using the three-feature-group approach compared to using the conventional feature set as a whole. Besides, the suggestion of placing the Attention network right after the BiLSTM network has made a lot of sense as this module helps the system to collect and highlight many important IP behaviors through the flow network.

Finally, the proposal to build and represent the relationships of IPs, and then use DGCNN to extract behaviors based on these relationships has added meaningful and valuable information in the behavior profile of the IPs, which then helps to improve the efficiency of the APT and normal IP classification process.

#### Threats to validity and future development direction

Firstly, this is about synthesizing and building behavioral profiles of IP based on the flow network. In this article, the BiADG model has built and synthesized much important information about IP by dividing flow characteristics into 3 main groups of characteristics. However, because the flow numbers of normal IPs and APT IPs are very different and disparate, improved techniques are needed at this stage to balance the flow numbers of each IP. This will help improve the IP classification process. CGAN, information gain are techniques that can be applied at this stage. Secondly, drawing from the experimental results of the BiADG model proposed in this paper, it can be seen that although the BiADG model has greatly helped improve the efficiency of the process of correctly classifying APT IPs in the unbalanced data, the experimental results are still relatively error-prone, i.e. there are more than 100 wrongly predicted APT IPs. Therefore, it can be noted that the BiADG model, although it has overcome the problem of IP APT attribute selection and extraction, but in the imbalanced datasets, this model still has some limitations. This means that it is necessary to have some further research on the BiADG model to deal with the data imbalance issue. Some techniques for data balancing can be applied such as Synthetic Minority Over-sampling, Generative Adversarial Networks, Dropout, etc. Accordingly, to improve the method, after extracting IP attributes through the flow network, instead of passing it through DGCNN, it first needs to be rebalanced through some data generation techniques such as Synthetic. Minority Over-sampling, Generative Adversarial Networks, Dropout, etc. The generated data can then be classified or further computational techniques performed. With this approach, there may be no need to use deep learning graph networks and data processing time can be greatly reduced. The novelty and uniqueness of this method is that it generates new data to help the labels be balanced, thereby improving the efficiency of the classification process. Finally, after the data is analyzed and extracted through the layers of the BiADG model, it is classified only through the softmax function in DGCNN, leading to limited results. In the future, to improve this classification, some advanced machine learning methods such as: representation learning and contrastive learning can be used. Representation learning model can be used by combining some deep learning algorithms such as CNN, MLP, and LSTM with some loss functions such as Contrastive Loss, Triplet Loss^53^, N-Pair Loss^54^, etc. For the contrastive learning method, some supervised or semi-supervised classification models can be used. We believe that applying these models is a very effective proposal for detecting APT IPs.

## Conclusions

This study has successfully proposed a new BiADG model for APT attack detection based on the combination of deep learning and Attention networks. With this flexible combination of networks, the newly created framework not only enables the high-accurate detection of APT attacks, but also reduces the rate of false alarm prediction. This shows that the BiADG model is not only meaningful in terms of scientific studies but also has many promising practical implementations. Based on the experimental scenarios to answer a variety of different questions, we have demonstrated the scientific validity and effectiveness of the BiADG Framework in the task of APT IP classification. The scientific validity of BiADG is reflected in the flexible combination of methods: (i) IP attribute synthesis through the flow network, (ii) APT IP attribute extraction. Finally, based on this proposal, we have successfully built a new Framework that provides good APT IP prediction results that are suitable for practical needs. The experimental results using this new Framework have once again demonstrated the arguments in section “Contribution of this paper” of the paper are entirely scientifically grounded and practically significant. With the approach of using BiADG to build and extract anomalous behavior of IPs from flow network, the BiADG model proposed in this paper is expected to not only identify APT attacks but also prove to be effective in addressing other cybersecurity issues, such as detecting botnets, DOS attack detection, DDOS, etc. In the future, this model can be further investigated for both APT and many other network anomalous activity detection. There are two main approaches to improve the APT IP detection model, including (i) rebalancing data, and (ii) representation learning. Applying these two methods help the system improve the accuracy of predicting APT IPs as well as normal IPs.

## Data Availability

The datasets and code generated and (or) analysed during the current study are available from the corresponding author on reasonable request. Replication package URL: https://github.com/nguyenthanhtung2?tab=repositories.
